# Natural and artificial feeding management before weaning promote different rumen microbial colonization but not differences in gene expression levels at the rumen epithelium of newborn goats

**DOI:** 10.1371/journal.pone.0182235

**Published:** 2017-08-16

**Authors:** Leticia Abecia, Elisabeth Jiménez, Gonzalo Martínez-Fernandez, A. Ignacio Martín-García, Eva Ramos-Morales, Eric Pinloche, Stuart E. Denman, C. Jamie Newbold, David R. Yáñez-Ruiz

**Affiliations:** 1 Estación Experimental del Zaidín (CSIC), Granada, Spain; 2 CSIRO, Agriculture & Food Flagship, Queensland Bioscience Precinct, St. Lucia, Queensland, Australia; 3 IBERS, Aberystwyth University, Aberystwyth, United Kingdom; Ben-Gurion University of the Negev, ISRAEL

## Abstract

The aim of this work was to evaluate the effect of feeding management during the first month of life (natural with the mother, NAT, or artificial with milk replacer, ART) on the rumen microbial colonization and the host innate immune response. Thirty pregnant goats carrying two fetuses were used. At birth one kid was taken immediately away from the doe and fed milk replacer (ART) while the other remained with the mother (NAT). Kids from groups received colostrum during first 2 days of life. Groups of four kids (from ART and NAT experimental groups) were slaughtered at 1, 3, 7, 14, 21 and 28 days of life. On the sampling day, after slaughtering, the rumen content was sampled and epithelial rumen tissue was collected. Pyrosequencing analyses of the bacterial community structure on samples collected at 3, 7, 14 and 28 days showed that both systems promoted significantly different colonization patterns (P = 0.001). Diversity indices increased with age and were higher in NAT feeding system. Lower mRNA abundance was detected in TLR2, TLR8 and TLR10 in days 3 and 5 compared to the other days (7, 14, 21 and 28). Only TLR5 showed a significantly different level of expression according to the feeding system, presenting higher mRNA abundances in ART kids. PGLYRP1 showed significantly higher abundance levels in days 3, 5 and 7, and then experienced a decline independently of the feeding system. These observations confirmed a highly diverse microbial colonisation from the first day of life in the undeveloped rumen, and show that the colonization pattern substantially differs between pre-ruminants reared under natural or artificial milk feeding systems. However, the rumen epithelial immune development does not differentially respond to distinct microbial colonization patterns.

## Introduction

Ruminants harbor a complex and diverse microbial ecosystem in their rumen that allows them to covert digested plant material into edible high nutritive quality products (meat and milk) [1]. At birth the rumen is not yet developed and functional. The process of bacterial colonization in the developing rumen is key for the achievement of rumen functions, which are a prerequisite for weaning and can thereafter affect efficiency and stability of digestion [2,3]. There is increasing evidence that populations established in early life of the animal may persist into later life, exerting physiological, metabolic and immunological effects, and offering potential of ‘programming’ the microbiome of the adult animal and potentially the animal’s performance [4,5]. However, the window of time in which the ecosystem is most sensitive to alteration is yet unknown and describing the sequential colonization in different feeding systems could help to design efficient intervention strategies to manipulate rumen microbial colonization in early life. Two main systems exist for rearing offspring in ruminant production. In commercial dairy systems, newborns are typically separated from the dam after birth and fed either milk replacer or whole milk; in contrast, in meat and extensive production systems, the offspring remains with the dam until weaning. These two systems therefore imply differences in regards to milk type (whole milk vs. milk replacer) and presence/absence of older companion, which can not be addressed separately.

Ruminant placenta impedes the transfer of Immunoglobulins (Ig) from the dam to the fetus, consequently, the consumption of colostrum by offspring has a fundamental role in the acquisition of passive immunity [6,7]. The Ig are the principal agents that protect the gut epithelium against pathogenic microorganisms, and IgG antibodies express multifunctional activities, including complement activation, bacterial opsonisation and agglutination, and act by binding to specific sites on the surfaces of most infectious agents or products, either inactivating them or reducing infection [8]. However, less clear is how the colonization of commensal microbiota interacts with the host immune system.

The ruminal epithelium is continuously exposed to commensal microbiota, pathogens and dietary antigens and provides a critical barrier between the host and the gut environment. Toll-like receptors (TLRs), peptidoglycan recognition proteins (PGLYRP1), and antimicrobial peptides (β-defensin) have been reported to interact with microbes to maintain gastro-intestinal homeostasis, including the rumen [9]. Toll-like receptors are present on a wide range of cells and can detect conserved molecular products of microorganisms [10]. It is important to understand host-microbiome interactions within the context of individual animal species and specific management practices. Data is now being generated revealing significant associations between the early microbiome, development of the gut immune system and the growth and health of newborns [11]. However, little information is available specific to the rumen ecosystem.

The aim of the present study was to evaluate the effect of feeding management during the first month of life (natural feeding with the dam, NAT, or artificial with milk replacer and isolated from adult animals, ART) on the sequential rumen bacterial colonization and the associated host immune response.

## Material and methods

All management and experimental procedures involving animals were performed by trained personnel in strict accordance with the Spanish guidelines (RD 1201/2005 of October 10, 2005) for experimental animal protection at the Estación Experimental del Zaidín (CSIC). Experimental protocols were approved (October 1, 2012) by the Ethics Committee for Animal Research at the Animal Nutrition Unit.

### Diets

Twenty eight pregnant Murciano-Granadina goats carrying two fetuses were selected and kept in individual pens (1.7 × 1.2 m) with free access to water. They were fed alfalfa hay *ad libitum* once per day (in g/kg of DM: OM, 880; CP, 214; ether extract, 13.6; NDF, 419: ADF, 244; and ADL, 61) and a supplement 600 g/d fed twice per day (0900 and 1500 h) based on (g/kg) wheat shorts (350), corn shorts (100), corn grain (50), barley grain (160), soybean hulls (90), soybean meal (90), sunflower meal (120), CaO (22), NaCl (3.5), calcium salts (4.5), and trace minerals and vitamins supplement (10; in g/kg of DM: OM, 893; CP, 170; ether extract, 33.9; NDF, 342; ADF, 142; and ADL, 34.3).

Kids had free access to the same hay and were offered *ad libitum* a commercial starter concentrate based on (g/kg): wheat shorts (50), corn shorts (50), corn grain (150), oat grain (260), milk powder (190), soybean meal (172), sunflower meal (120), NaCl (3.5), and calcium salts (4.5; in g/kg of DM: OM, 925; CP, 162; ether extract, 35; NDF, 163; and ADF, 78).

### Experimental design

The experimental period commenced after parturition. All does gave birth to two kids, one of which remained with the dam (NAT) while the other was taken immediately away and fed colostrum for 48 hours and milk replacer (ART) (Sello azul, Lemansa, León, Spain). Colostrum from each goat mother was fed to both of her twin kids. Groups of four kids (from both ART and NAT experimental groups) were randomly selected and slaughtered at 1, 3, 5, 7, 14, 21 and 28 days of life. On the sampling day blood samples were taken for Ig quantification in plasma. After slaughtering, rumen content was sampled and aliquots immediately stored at -80°C for volatile fatty acids (VFA) [4] and molecular analyses. Samples of the rumen epithelium (approximately 500 mg) were biopsied, from the ventral sac and washed immediately after collection with 0.01 M phosphate-buffered saline (PBS) buffer (pH 6.8). The samples of cleaned tissue were then transferred to RNA-later solution (Qiagen Ltd, West Sussex, UK) and stored at −80°C until further analysis. Rumen digesta samples from day 1 contained very low volume and could only be used for VFA analyses.

### Pyrosequencing and sequence analysis

Total DNA was extracted from 0.5 g of each sample collected on days 3, 7, 14 and 28 using QIAGEN QIAamp^®^ DNA stool mini kits (Qiagen Ltd, West Sussex, UK). The yield and purity of the extracted DNA were assessed using NanoDrop^®^ ND-1000 Spectrophotometer (NanoDrop Technologies, Wilmington, USA). Amplification of the bacterial V1-V3 regions of 16SrRNA was performed using the primer pair 27F and 533R (27F 5' GTT TGA TCC TGG CTC AG 3' and 533r 5' TTA CCG CGG CTG CTG GCA C 3'). Primers incorporated 10nt barcode tags allowing samples to be multiplexed and Roche/454 adaptors. PCRs were performed, per triplicate, in a total volume each one of 25 μL containing 10x PCR buffer, 10 mM dNTP mix, 10 pmol/μL of forward and reverse primers, 1U FastStart Polymerase, and 1 μL of DNA template. The amplification conditions were: an initial denaturation step at 95°C for 2 min; 30 cycles of denaturation at 95°C for 30s, annealing at 55°C for 30s, and elongation at 72°C for 2 min; and a final extension step at 72°C for 7 min The size of the PCR products was then checked on a 1% agarose gel electrophoresis. Following this, triplicates were pooled together and products were then purified using the short fragment removal method described by Roche using their GS FLX amplicon DNA preparation guide and AMPure beads. The purified PCR products were quantified using Quant-iT PicoGreen dsDNA quantification kit (Invitrogen) and mixed in equimolar amounts to 10^7^ molecules/ μL sample. The amplicon pooled libraries were pyrosequenced on a Roche 454 FLX Titanium.

### Sequence preprocessing and statistical analysis

Short read sequence data generated was analyzed using QIIME: Quantitative Insights Into Microbial Ecology software package [12]. Raw sequences were passed through Acacia for 454 error correcting [13]. Error corrected sequences were then de-multiplexed in QIIME based on their unique barcode, clustering of sequences to OTUs of 97% similarity were performed using closed-reference OTU picking method uclust [14]. Taxonomic assignment of sequences was performed against the Greengenes database [15] using the RDP classifier software [16]. Alpha and beta diversity and significant change of OTU’s were performed in the R packages ade4, Phyloseq, and vegan [17,18,19]. The significances of grouping in the PCoA plots were tested by analysis of dissimilarity (ADONIS) with 999 permutations. The sequences obtained in this paper were deposited in the European Nucleotide Archive (ENA) under the project number PRJEB20457.

### RNA extraction and gene expression quantification

Total RNA samples were extracted from 50–100 mg of rumen epithelial samples. They were homogenized with 0.9 mm stainless steel bead and 1 ml of TRIzol Reagent (Invitrogen, CA, USA), using a Bullet Blender^®^ homogenizer (Next Advance, Inc. NY, USA). The homogenate was incubated for 5 min at RT to allow dissociation of nucleoprotein complexes before adding 200 μL chloroform mL^–1^ of TRIZOL containing tissue homogenate. The solution was mixed briefly, incubated for 3 min at RT, and then centrifuged at 12 000 × g for 10 min at 4°C. The aqueous phase was collected, precipitated with 500 μL isopropanol mL^–1^ of TRIZOL containing tissue homogenate, incubated for 5 min at RT and then applied to an RNeasy Mini-column (Qiagen, UK) before spinning for 15 s at 8000 × g. The flow through was discarded and RNA bound to the column was DNase treated using RNase-Free DNase Set (Qiagen) and collected in a 50 μL volume. The RNA quantity was measured using ND 1000 spectrophotometer (NanoDrop Technologies, Wilmington) and RNA integrity number was measured using Bioanalyzer 2100 (Agilent Technologies, Santa Clara, CA).

The extracted total RNA was reversed transcribed using QuantiTect Reverse Transcription Kit (Qiagen, UK) according to the protocol. The RNA was used as template for qRT-PCR analysis to evaluate the expression of genes encoding 10 bovine TLR, β-defensin and peptidoglycan recognition protein 1 (PGLYRP1) in the rumen epithelium using the gene specific primer pairs (S1 Table) relative to β-actin expression. For each PCR reaction, 25 ng of cDNA was amplified with each primer set using the following parameters: 55°C for 2 min to eliminate carry-over dUTP, 95°C for 8.5 min, then 45 cycles of 95°C for 15 s; 58°C for 30 s; and 72°C for 30 s on the Bio-Rad iCycler (Bio-Rad Laboratories Ltd., Mississauga, ON). Amplification data are expressed as change in Cycle threshold (ΔCt) and calculated as follows: (ΔCt = Cycle threshold of Gene of Interest—Cycle threshold of β-actin). A smaller ΔCt value equates to more abundant transcript.

### Quantification of immunoglobulins

Plasma samples were centrifuged at 3000 × *g* for 5 min at 4°C just before the analysis. Immunoglobulin A and G were measured from plasma using an ELISA quantification kit from Bethyl Laboratoires (Montgomery, TX), following the manufacturer´s instruction. IgG determination in plasma samples were determined between 1:150000 and 1:50000 dilutions for 3–7 d and 14-28d, respectively. IgA determination in plasma samples was determined between 1:1000 and 1:200 dilutions at 3 and 14 or 28, respectively. Data were expressed as mg or μg per millilitre of sample for IgG or IgA, respectively. Duplicate determinations were performed on each plate.

### Statistical analyses

Data were analyzed using the SAS PROC MIXED procedure (SAS Inst. Inc., Cary, NC). The statistical model used included the effects of feeding management to kids, the age and the feeding management × age interaction as fixed effects. Animal effect was considered random. When feeding management × age interaction was significant (*P* < 0.05), differences between treatment means were evaluated using the pdiff option of the LS means statement in the MIXED procedure of SAS and declared significant at *P* < 0.05. A tendency (T) was considered when *P*-values were <0.1.

## Results

### Bacterial community

**Fig 1:**
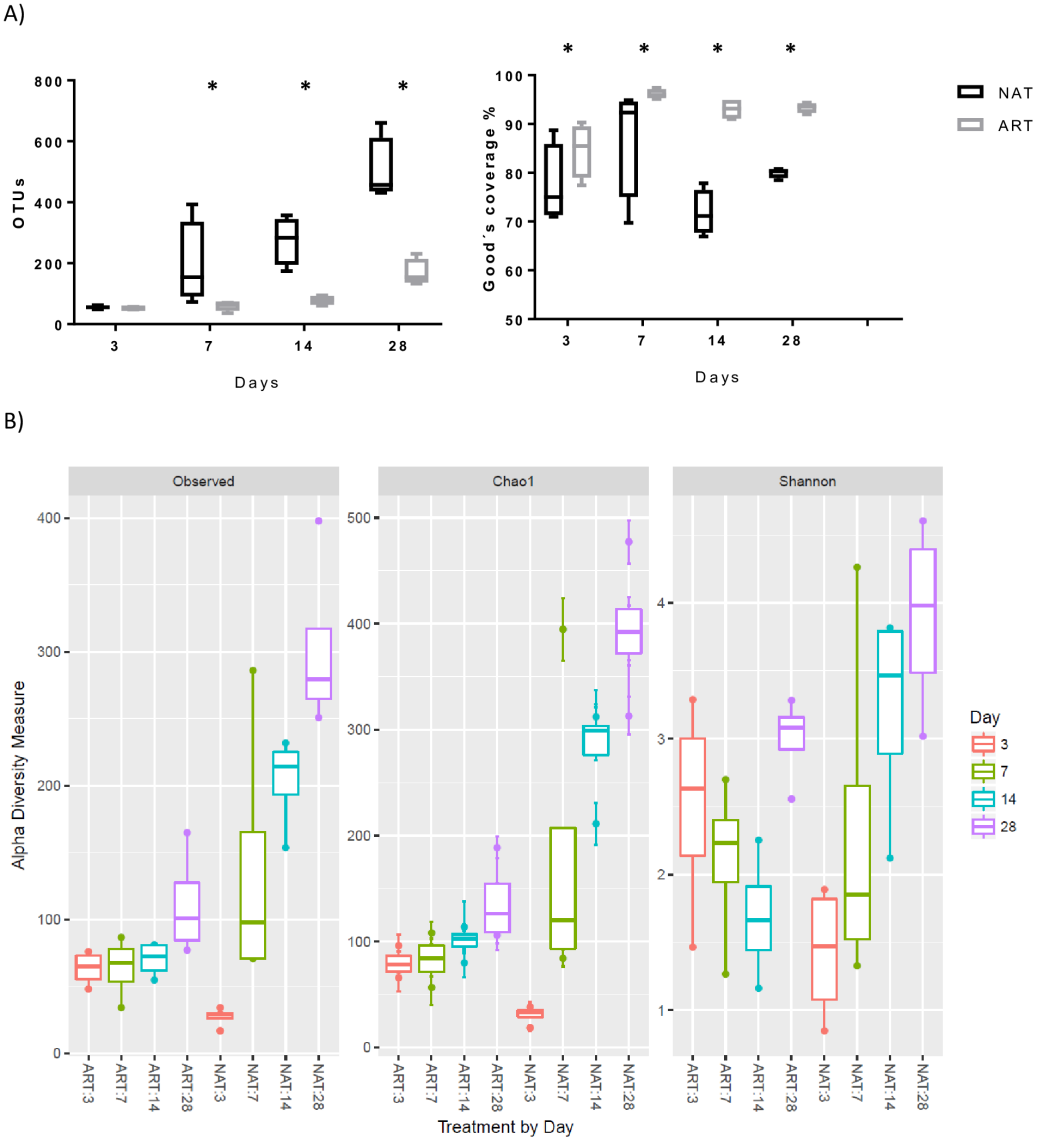
Alpha diversity measures for rumen bacteria across different feeding system the first month of life. Total Observed taxonomic units, Chao1 estimates and Shannon diversity index. Boxplots indicate the first and third quartiles with the median value indicated as a horizontal line the whickers extend to 1.5 times the inter quartile range. NAT, natural; ART, artificial feeding management.

A total number of 141197 reads, with an average of 4412 ± 2464 sequence reads were observed per sample. The average length of sequence reads after primer removal was 470 bp. The overall number of OTUs detected by the analysis reached 1556, based on 97% nucleotide sequence identity between reads and increased with age. Rumen contents from NAT kids presented greater number of OTUs than in ART kids from day 7 (Fig 1A). The bacterial diversity (measured as different indexes, Fig 1B) increased significantly with age and was greater in NAT kids.

### OTU diversity and similarity analysis

**Fig 2:**
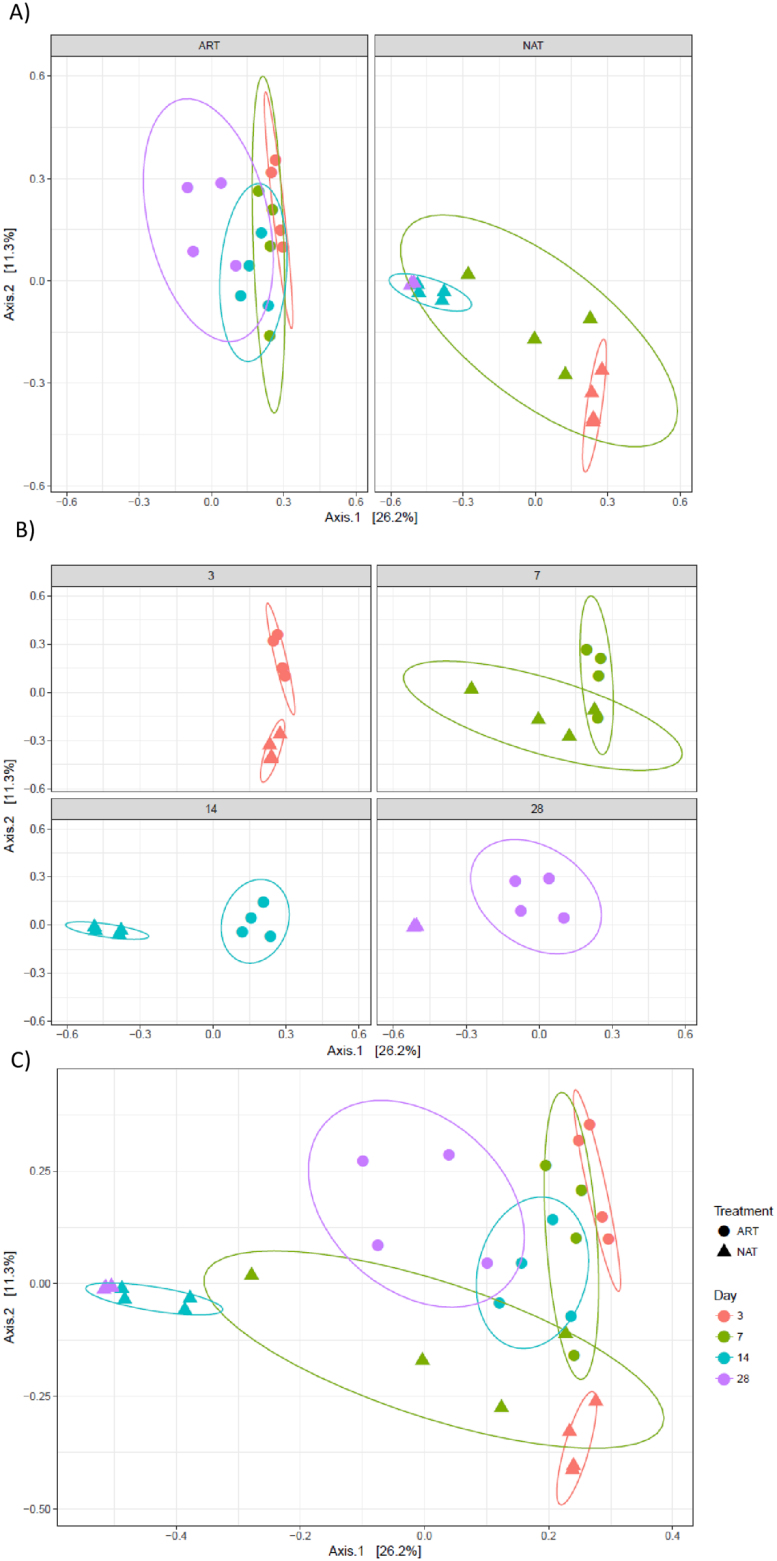
Principal coordinate analysis (Bray Curtis distance) comparing changes in rumen bacterial community. (A) age within feeding system (NAT or ART). (B) feeding system within each age (3, 7, 14 and 28 days). (C) age and feeding system.

When assessing the data by principle coordinate analysis, a clear pattern was observed according to feeding management (Fig 2A) (P = 0.001) and age (Fig 2B). Pairwise comparison between different ages of the animals showed that day 3 presented a significantly different bacterial community (P = 0.001). Clear differences between the community at days 7 and 28 were found (P = 0.004).

### Taxonomic composition of ruminal bacterial community

Overall, the abundance of 17 phyla was detected in rumen content samples, these were *Actinobacteria*, *Bacteroidetes*, *Chloroflexi*, *Cyanobacteria*, *Elusimicrobia*, *Fibrobacteres*, *Firmicutes Fusobacteria*, *Lentisphaerae*, *Proteobacteria*, *Spirochaetes*, *SR1*, *Synergistetes*, *Tenericutes*, *TM7*, *Verrucomicrobia and WPS-2*. Among them, *Proteobacteria*, *Firmicutes* and *Bacteroidetes* were identified as the dominant phyla regardless of the age group, but their ratio and composition differed considerably between NAT and ART kids. In both experimental groups *Bacteroidetes* and *Proteobacteria* were the two main phyla that colonized the rumen at day 3, reaching proportions as high as 73 and 67% of total sequences, for NAT and ART, respectively. Afterward, the relative abundance of *Proteobacteria* declined rapidly with age. In contrast, *Firmicutes*, *Fibrobacter and Cyanobacteria* increased with age significantly. The *Fusobacterium* peaked at day 7 (P = 0.03). The phylum *Bacteroidetes* was the dominant from day 7 onwards in both feeding managements. The relative abundance of *Spirochaetes* phylum was significantly higher in NAT feeding system (P = 0.014).

**Fig 3:**
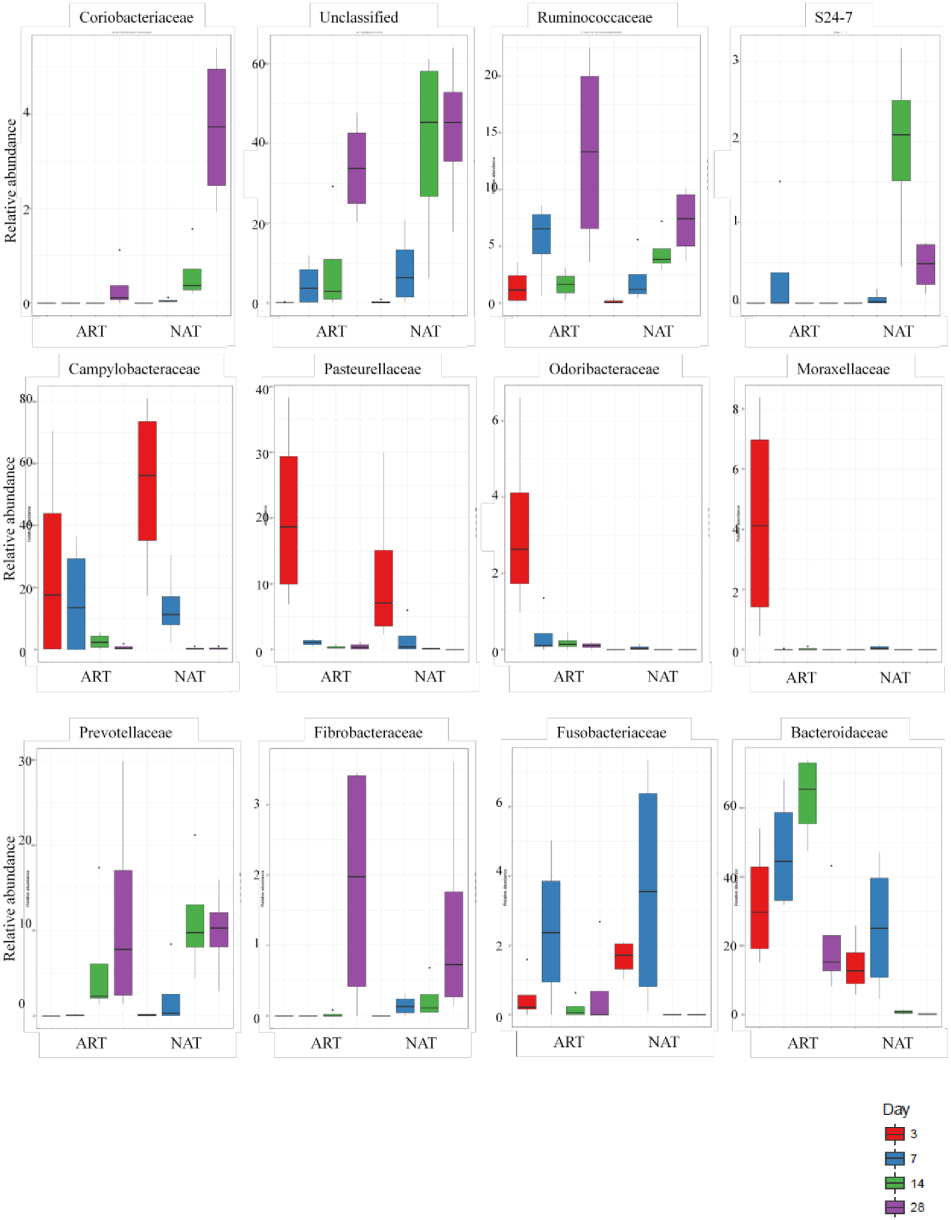
Family level taxonomic composition of the ruminal bacterial community at different ages of kids (% of sequences).

Of the 88 families detected, 15 changed significantly over the experimental period (Fig 3). The *Coriobacteriaceae*, *Ruminococcaceae*, *S24-7*, *Prevotellaceae*, *Fibrobacteraceae* and *Alcaligenaceae* families increased with time. Similarly, unclassified sequences abundance increased with age (P = 0.001). Conversely, *Campylobacteraceae*, *Pasteurellaceae*, *Odoribacteraceae and Moraxellaceae* abundance decreased with time. The abundance of *Bacteroidaceae* family was affected by feeding system (P = 0.0001). The effect between feeding system and age modified *Coriobacteriaceae* and *S24-7* families, being more abundant in NAT feeding as animals were older.

Of the 132 genera detected, 17 were over 0.1% relative abundance and changed significantly over the experimental study (S1 Fig). The *Aggregatibacter*, *Actinobacillus*, *Arcobacter*, *Acinetobacter*, *Butyricimonas*, *Haemophilys* and *Mannheimia* relative abundance decreased very rapidly with age. In contrast, *Fibrobacter*, *Moryella*, *Mogibacterium*, *Prevotella*, *Ruminococcus*, *Succiniclasticum*, *Sutterella* and *TG5* genera increased with age. The *Bacteroides* and *Fusobacterium* were dominant in their families and followed the same pattern as described for the family, similarly to unclassified sequences. The genus *Moryella*, *Bacteroides* and *Succiclasticum* were affected by feeding system, *Bacteroides* being higher in ART (P = 0.0002) and *Succiniclasticum* (P = 0.03) in NAT feeding system. The relative abundance of *Moryella* and *TG5* genus was significantly higher in NAT feeding system with older kids (P = 0.0003 and P = 0.03, respectively). On the contrary, *Actinobacillus* genus peaked at day 3 in the rumen of ART kids (P = 0.03).

### TLRs, β-defensin and PGLYRP1 expression

The expressions level of genes of interest in the rumen epithelia of kids were measured using qRT-PCR relative to β-actin expression (Malmuthuge et al., 2010) using the ΔC_t_ value. Expression of the 10 TLR was detected from day 3 (Table 1). The lowest expression levels corresponded to TLR 2 and 5, while the highest was TLR6. Lower mRNA abundance was detected in TLR2, TLR8 and TLR10 in days 3 and 5 compared to the other days (7, 14, 21 and 28). Only TLR5 showed a significantly different level of expression according to the feeding system, presenting higher mRNA abundances in ART kids. PGLYRP1 showed significantly higher abundance levels in days 3, 5 and 7, and then experienced a decline independently of the feeding system.

**Table 1:** Expression of 10 TLRs, β-defensin and peptidoglycan recognition protein 1(PGLYRP1) in rumen of kids at 3, 5, 7, 14, 21 and 28 days old fed on two feeding systems (Natural and Artificial). ΔC_t_ = C_t (TLRs)_ − C_t (βactin)_, lower ΔC_t_ represent higher mRNA abundance level and *vice versa*.

Feeding	Age	TLR1	TLR2	TLR3	TLR4	TLR5	TLR6	TLR7	TLR8	TLR9	TLR10	β-defensine	PGLYRP1
**Natural**	3	11.52	23.92	12.01	9.64	22.34	9.95	10.81	15.04	11.77	17.00	16.78	10.81
	5	11.51	24.19	12.79	10.53	24.03	9.58	11.18	14.88	12.21	16.60	15.58	10.74
	7	10.11	18.90	10.89	9.32	23.97	8.76	9.12	11.73	10.31	14.31	15.80	10.58
	14	11.40	20.36	10.85	10.58	25.22	8.61	12.42	12.76	13.17	15.04	17.63	15.44
	21	11.31	ND	11.95	10.71	23.69	10.40	12.64	13.05	11.33	15.21	15.11	15.48
	28	11.87	19.62	10.66	10.33	22.15	9.07	9.71	13.10	10.47	15.71	15.38	14.53
**Artificial**	3	10.48	24.79	10.53	9.99	20.78	8.59	10.95	14.45	11.45	16.47	15.79	10.70
	5	10.58	24.74	10.94	10.15	20.86	8.88	10.98	14.07	12.17	16.78	15.21	10.44
	7	11.07	19.97	11.44	10.18	23.72	8.86	10.54	13.41	11.51	15.89	15.33	11.31
	14	11.62	18.99	11.86	10.68	22.63	9.01	10.31	13.50	11.18	15.82	15.87	11.81
	21	11.28	ND	10.72	10.15	23.04	9.23	10.57	13.21	11.08	16.15	15.00	14.49
	28	11.64	20.19	10.68	10.41	23.23	9.43	10.60	13.89	11.37	15.68	15.10	14.89
	SED	0.638	1.121	0.916	1.092	1.384	0.612	1.814	1.122	1.487	0.831	1.249	1.721
**P-value**	Feeding	0.47	0.49	0.13	0.88	0.0235	0.078	0.61	0.41	0.87	0.10	0.13	0.28
	Age	0.081	<0.0001	0.39	0.51	0.06	0.15	0.54	0.0201	0.52	0.0108	0.22	<0.0001
	Interaction	0.18	0.62	0.099	0.85	0.16	0.065	0.49	0.43	0.56	0.29	0.89	0.39

SED: standard error of difference.

ND: Not determined

### Immunoglobulin concentration

The IgG levels in plasma (Table 2) were affected by feeding system and age, being higher the first 7 days of age in NAT group compared to ART, and then became progressively similar from day 14 onwards. Feeding system did not have any effect on IgA levels in plasma of kids; however the titers were highest on days 1 and 3 and started declining from day 5 onwards.

**Table 2:** Immunoglobulin A and G were measured from plasma at different ages and feeding systems in kids. Results were expressed as mg/ml and μg/ml for G and A, respectively.

Feeding	Age	IgG	IgA
**Natural**	1	27.4^a^ ^A^	157.5^A^
	3	20.9 ^A^	181.3^A^
	5	10.8 ^B^^C^	122.1^B^
	7	14.5^a^ ^B^	82.4^B^^C^
	14	4.5 ^C^	16.8^C^
	21	3.8 ^C^	16.7^C^
	28	3.5 ^C^	3.7^C^
**Artificial**	1	15.8^b^	192.2
	3	17.01	187.9
	5	5.9	92.0
	7	8.9^b^	104.7
	14	5.9	95.5
	21	3.8	9.7
	28	3.8	6.7
	SED	3.039	28.76
**P Value**	Feeding	0.002	0.254
	Age	<0.0001	<0.0001
	Feeding x Age	0.034	0.504

SED: standard error of difference.

^a–c^Within a column, means without a common superscript letter differ according to feeding;

^A–C^Within a column, means without a common superscript letter differ according to age, p ≤ 0.05 (LSD test).

## Discussion

This experiment used the two main offspring rearing systems in ruminant livestock to assess whether this differential management exerted any effect on rumen bacterial colonisation and innate immune response. The aim was not to compare the effect of two milk types (natural vs. milk replacer) with inherent differences in composition, but the whole management system, which also accounts for presence/absence of the dam. A previous work from the same experimental trial [20] described the effects on bacterial, protozoal and archaeal biomass colonization, rumen development and fermentation. The results confirmed substantial microbial colonisation from the first day of life in the undeveloped rumen. Moreover, the concentration of bacteria was higher in the rumen of NAT kids on days 3, 5, 7 and 14 and of protozoa from day 3 onward. The objective of the present work was to complement the previous observations by describing the sequence of colonization of bacterial groups to confirm a differential pattern between two feeding systems and the link with the host immune response.

### Rumen bacterial community composition and diversity indices

In ruminants, the gastrointestinal tract, including the forestomach complex, is sterile at birth but it is rapidly colonized after. Previous work emphasizes the essential role that the dam plays as a ‘microbial inoculator’ [21] from several sources: vaginal canal, fecal material, colostrum, skin and saliva. The primo-colonizing bacteria may play an important role in shaping the biotope for strictly anaerobic microbial populations colonizing rapidly afterward [22]. Our results are consistent with those reported recently [23–26], microbial diversity and species richness increased over time when microbiota becomes more mature and stable as the animal is aging. In the NAT feeding system group, diversity was higher and the community structure different from ART group in which animals were separated from the doe after birth.

Based on the current analysis, the dynamic of the bacterial community establishment in kids could be divided in three steps, including a progressive change in the bacterial community composition. The **first** 2–3 days of life correspond to the initial phase of colonization corresponding to the pioneer colonizing bacteria [27] and the profile of bacterial community was completely different from all the later ages, as it was described at day 2 by Rey et al. [28]. Bacteria belonging to *Proteobacteria*, *Bacteroidetes*, *Firmicutes*, *Actinobacteria* and *Fusobacteria* phyla were present at this stage, and the *Proteobacteria* phylum represented more than 50% of total bacteria. These phyla comprise both obligate and facultative anaerobic bacteria genera, which is consistent with other authors [28,29,30] who also observed a large proportion of both facultative and strictly anaerobic bacterial communities between 2 and 3 days of age of ruminants. In goat kids, Jiao et al. [20] noted that on the day of birth, measurements of the phylum *Proteobacteria* far exceeded other phyla (90.13%), with the majority belonging to the genus *Escherichia* (80.8%) that comprises facultative anaerobic bacteria. This microbiota might be derived from that of the mother’s vagina, skin, colostrum, or nearby environment, and may have a particular importance in scavenging oxygen diffusing from the capillary network, thereby creating ecological conditions suitable for the establishment of anaerobic communities [31]. In our work, *Arcobacter* was present in high proportion during the first week of life in agreement with Jiao et al. [25], who found this genus in the rumen epithelial bacteria community of goats at the first week of life. *Arcobacter* is aerotolerant and the majority of these bacteria are oxidase-positive microaerophiles indicating an electron transport chain with molecular oxygen as terminal electron acceptor [32].

In the **second** stage of colonization kids switched from colostrum to mother´s milk (NAT) or milk replacer (ART). Jiao et al. [25] observed remarkable changes in ruminal epithelial bacterial communities during the first week after birth with a rapid change in the rumen environment as it was observed in our study in rumen content from goats. This change was probably due to the ‘pioneer’ community that allowed the establishment of other bacteria and plays an important function in the early maturation of the microbial community. Thus changes in the rumen environment were probably related to a switch from aerobic or facultative anaerobic to strictly anaerobic bacteria, as previously described by Jami et al. [23] in colostrum fed calves. After day 3, the bacterial colonization presented large differences in the community assemblies among individual kids, supporting the concept of a interaction between the microbiota and the host [28,33,34].

**Fig 4:**
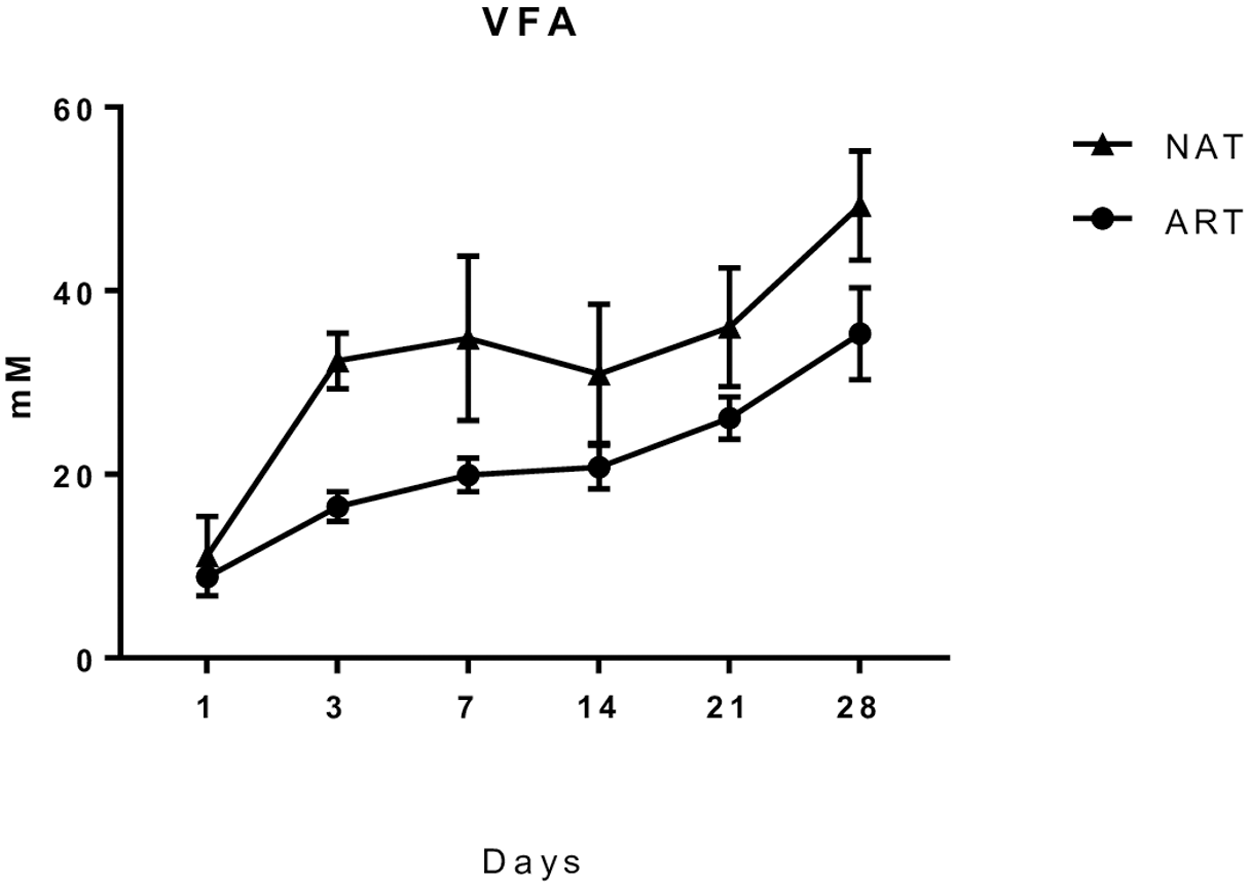
Total VFA concentration in the rumen of kids fed on natural or artificial systems.

The **third** stage of colonization covers from day 14 to 28 or weaning. During this stage, starter concentrate intake (and some small pieces of forage) progressively increased their proportion in the diet and bacterial community presented a fluctuation according to two main elements: feeding behavior and probably the presence/absence of adult animals. From day 14, the community no longer exhibited clear time-related changes. As noted earlier, at this stage, the concentrate intake by kids increased and this was reflected in an increase in VFA concentration (Fig 4). It has been suggested that the earlier the animals start consuming solid feed, the quicker their rumen bacterial community becomes more similar to that in adults, probably selecting bacterial microbial taxa that are more specific and adapted to new substrates [35].

Jami et al. [23] reported that the matured bacterial community was not detected before month 6 of age, when *Bacteroidetes*, *Firmicutes*, and *Proteobacteria* were among the dominant phyla. However, more recently it has been reported that the colonization by rumen epithelial bacteria is age-related and achieved at 2 months of life [25]. In agreement with previous studies in cattle [23,35], our results suggest that the developing rumen comprises the same dominant phyla as that in the more mature, although the relative abundances vary with stage of development and diet. In our study, *Bacteroidetes*, *Firmicutes*, and *Proteobacteria* accounted for a total of 84.6 and 86.9% in the rumen of NAT and ART feeding group at day 28, respectively, *Bacteroidetes* being the dominant phylum. This in agreement with previous studies who reported that *Bacteroidetes* was the most abundant phyla from days 19 to 83 in pre-weaned calves [28] and that the same three phyla were dominant regardless of the age group (0–70 d) in rumen epithelial bacteria [25]. The abundance of *Proteobacteria* decreased while the abundance of *Bacteroidetes* and *Firmicutes* increased with age as it has been also detected in rumen epithelial bacterial diversity in goats [25]. This phylum was dominated by the g. *Prevotella* despite decreasing in abundance in NAT group. The dominance of g. *Prevotella* in the mature ruminal community has been reported previously in cattle [36,37,38] and in recent international survey of the rumen and foregut microbial communities in 742 samples collected from 32 ruminant species across 35 countries from different geographical regions [6] reported that the most abundant and prevalent taxa within the rumen microbiota was *Prevotella* genus. The *Bacteroides* genus was present in high abundance in the rumen of ART group from day 3 to 28. Our results indicate that *Bacteroides* is linked to the consumption of high caloric milk replacer in pre-weaned kids, as it was also described in pre-weaned calves by Meale et al. [35]. They linked the carbohydrate composition of the diet during the transition from milk replacer to starter with changing the ratio of Bacteroidetes to Firmicutes in the rumen.

With regards to the bacterial colonization pattern in NAT vs. ART kids, some differences were detected. *Fibrobacter* (one of the main cellulolytic phyla in the rumen) was present in NAT group at day 7 onward; however it did colonize the rumen of ART group until day 28, similarly to *Succiniclasticum* which ferment succinate to propionate. However, the abundance of these phylums is highly variable among individual animals and diets [6], this positively correlated with concentrate intake and VFA concentration. The greater VFA concentration observed in the rumen of NAT kids may suggest a relationship between the abundance of *Moryella* sp. with total VFA production as the major metabolic end product are acetic, butyric and lactic acids. However, it remains unknown whether the distinct bacterial colonization between animals reared naturally or artificially may have effects on the animal digestive performance later in life.

The differences observed in rumen bacterial colonization between NAT and ART kids may be ascribed to different factors: intake pattern of solid feed and the presence (NAT)/absence (ART) of the dam as inoculator of rumen bacteria through saliva, faces, etc… De Paula Vieira et al. [39] observed that the presence of an older companion with pre-weaned calves stimulated feeding behavior and growth before and after weaning from milk. A differential pattern in solid feed intake between NAT and ART kids may have provided a different substrate to ferment and therefore different bacterial groups promoted. The pattern of VFA concentration in the rumen as animals aged (Fig 4) suggests that NAT kids had greater feed intakes than ART ones. This is supported by results from De Paula Vieira et al. [39], which showed that calves reared in the presence of older companions had more frequent and longer visits to the feeder, which could be a result of social learning [40]. Anderson et al. [41] showed that introducing solid feed for early weaning (3 weeks) in calves promoted greater microbial abundance in the rumen as compared to calves weaned conventionally (6 weeks), but no assessment of the composition of the microbiota was performed. Early studies [42,43] reported that giving forage or forage plus concentrate around weaning determined the concentration of some anaerobic bacteria (lactobacilli and lactate-utilizing cocci).

The second factor that may promote a different colonization according to feeding management is the presence of dam and the associated increased in the availability of microorganisms in the environment, allowing earlier (and different) inoculation of microbes in the digestive tract of the NAT kids as compared to ART ones. Previous work using twins emphasized the essential role that the dam plays as a ‘microbial inoculator’ [21]. A direct contact with the mother offers a constant source of microbes through mouth, faeces, skin and milk, which are not available for the artificially reared kids. This would explain the greater number of OTUs and bacterial diversity observed in NAT kids. Another distinctive feature between NAT and ART kids was the almost absence of protozoal biomass in the rumen of artificially reared kids, as protozoa can only be inoculated in the rumen by direct contact with adult animals through saliva [20]. Protozoa are not essential for the normal rumen functioning [44]; however, the presence/absence of protozoa has been associated with the structure of different bacterial and methanogens communities and different rumen fermentation pattern [45,46]. For example, rumen protozoa have been associated with greater butyrate concentration [47], and butyrate is one of the main energy sources of epithelial cells and therefore promotes the development of the rumen papillae absorptive area [48], which could represent a clear advantage to overcome the stress associated with transition from liquid to solid diet at weaning. Indeed, supplementing butyric acid to young animals during pre-weaning has been shown to adapt rumen to postpartum diet and accelerate rumen development [49–50]. Although it has been shown that inoculation of protozoa later in life (either artificially or by grouping young animals with faunated ones) is possible, it remains unknown whether the indirect effect on the microbial community persists later in life [2].

### TLRs, β-defensin and PGLYRP1 expression

TLRs comprise a family of pattern-recognition receptors that detect conserved molecular products of microorganism, such as lipolysaccharide (LPS) and lipoteichoic acid (LTA). Viruses, bacteria, fungi and protozoa display several different molecular patterns that are recognized by different TLRs [51]. In the present study, TLRs 1, 2, 5, 8 and 10 displayed an age-dependent expression. The general pattern involved an increased in gene expression between days 5 and 7 and then a subsequent decrease and stabilization. The increase in expression coincides with the sharp raise in VFA concentration and microbial biomass colonization. However, although colonization continued in following days, this did not trigger higher expression levels. This is in agreement with Malmuthuge et al. [9] that reported an age-related down-regulation of the expression level of TLRs in the gastrointestinal tract of calves. Likewise, Teran et al. [52] found that the TLR expression levels in the blood of infants were down-regulated with increasing age, while memory of T cells increased in number. These changes are consistent with a decrease in innate immune responses that is balanced by an increase in adaptive immune response with increasing age. Down-regulation with increasing age has been suggested as one mechanism by which the host avoids unnecessary inflammatory responses to commensal microbiota [11] and our results support such hypothesis. TLR2 play very important roles in the host response to the cell wall components of pathogenic gram-positive bacteria, which can directly affect gut health [53] and also recognizes another component, bacterial lipopeptide, alone or cytoplasmic domain of TLR2 can form functional pairs with TLR1, TLR6 or TLR10 [54]. Interestingly, the two main phyla that colonized the rumen (*Bacteroidetes* and *Proteobacteria*) are mainly gram-negative. However with age, proportions of colonizing groups changed: *Proteobacteria* decreased and *Firmicutes* increased, most of which are gram-positive. Our results suggest that variations in bacterial population with time may represent variations in bacterial ligands which could play an important role in modulating TLRs expression and innate mucosal immune responses. Although TLRs do not, as a rule, exhibit specificity for a single microbial product, they are individually responsive to a limited group of molecules.

The observed increased in TLR10 expression as rumen developed might be an indication of variation in the gut maturity of animals, suggesting that higher expression of TLR10 might be very important in early epithelial immune system development. As TLR10 is a link between adaptive and innate immune responses [55], further studies on TLR10 and its functions in the gastrointestinal tract may lead to better understanding of the role of TLR10 in gastrointestinal tract health.

The expression of TLR5 was the only one that significantly differed between feeding systems, although mostly in the first week of life. TLR5 recognises bacterial flagellin, a principal component of Gram-positive and Gram-negative bacterial flagella. The interaction between TLR5 and flagellin also leads to the expression of antiapoptotic genes that are correlated with the protective effect of the receptor against normal commensal such as *E*. *coli* [56].

In an attempt to identify links between TLR expression and microbial colonization, we assessed potential correlation with the abundance of different bacterial groups and no significant relationships were observed (data not included). Apart from differences in bacterial groups abundance, the main feature that can distinguish NAT and ART groups is the presence/absence of protozoa. Unlike bacteria and viruses, protozoans often differentiate within the host into discrete forms that are morphologically and molecularly distinct. Recognition of protozoan pathogens by the host innate immune system presents further challenges, because, like their hosts, they are eukaryotic organisms. Nevertheless, protozoan-associated molecular patterns exist that are recognized by TLRs. These include dominant surface glycolipids (glycosylphosphatidylinositol anchors that are recognized by TLR2 and TLR4) and genomic DNA that activates TLR9 [57]. However, none of these three TLR showed a significant (P > 0.05) correlation with protozoan biomass (r = -0.12, 0.28 and 0.02, respectively for TLR 2, 4 and 9), suggesting that symbiotic protozoa can occupy ecological niches in the rumen without triggering a specific immune response at epithelium level.

The β-Defensin and PGLYRP1 are secretory antimicrobial defense molecules that are capable of recognizing and killing of pathogens [58]. Peptidoglycan recognition protein 1 mainly kills gram-positive bacteria however bovine PGLYRP1 has an affinity to kill fungi, and neutralize lipoteichoic acid, and lipopolysaccharides as well [59]. Peptidoglycan recognition protein 1 is exclusively expressed in polymorphonuclear leukocytes [59], suggesting the need for microbial products to reach into the epithelial tissue to activate PGLYRP1. Our results showed a marked decreased in PGLYRP1 levels of expression as rumen matured and no effect of feeding system. Malmuthuge et al. [9] observed very low expression of PGLYRP1 prior weaning and higher at 6 months of age in calves rumen. The different temporal sequence between our work and that of Malmuthuge makes comparisons very difficult. It has been noted that the first five days of life there is different gut permeability to allow immunoglobulins ingested from colostrum and allows more bacterial products to reach the mucosal barrier and stimulate the expression of host pattern recognition receptors [11]. This fact could be related with the higher abundance of mRNA the first week of life.

### Plasma immunoglobulins concentration

Although colostrum contains several types of immunoglobulins (IgG, IgA, IgM), IgG constitutes approximately 85% of the total, therefore, measurement of kid plasma IgG level is the standard for determining the level of passive transfer of immunity. Failure of passive transfer has been associated with decreased first and second lactation milk production and an increased culling rate during the first lactation [60–61].

Despite ART kid goats being fed the same colostrum as NAT kids during the first 48 hours of life, NAT group presented higher IgG levels due to continuous access to the mother´s udder for 24 hours. Differences due to feeding systems were observed only during the first week of life. IgG levels decreased from day 5 and values were stable from day 14 onwards. This agrees with Guidry et al. [62] who reported that in the transition from colostrum to mature milk, Ig levels decrease sharply during the first five days post-partum. The ruminant´s gastrointestinal tract is designed to temporarily allow the absorption of large molecules, including immunoglobulins, during the first 12 to 24 h of life. After this period, intestinal closure is produced [63]. Contrary to IgG, IgA levels did not differ between experimental groups. IgA in plasma provides the source for secretory IgA via gut epithelia or through saliva, which in ruminants represent the main vehicle of introducing immunoglobulins into the rumen [2]. Subharat et al. [64] recently showed that IgA resist longer in the rumen to degradation than IgG, due to the secretory component of IgA which makes the immunoglobulin more resistant to proteases [65].

In this experiment IgA levels were highest during the first 3 days of life and then declined sharply from day 14. There is ample evidence that primary gut microbial colonization after birth provides the antigenic stimulus for development of IgA responses [66]. Early work by Sharpe et al. [67] highlighted the strong link between rumen microbial colonization and specific antigen production. Unfortunately, after molecular techniques developed to study microbial ecosystem no further work has been conducted in ruminants reared under different conditions in early life. In our work, animals experienced different microbial colonization patterns, mainly, higher bacterial and protozoal biomass and more diverse bacterial community in NAT than in ART kids. However, this different pattern was not reflected in alterations in total IgA concentration in plasma. Further studies could address the response in secretory IgA in saliva, and paying attention on specific IgA in relation to the groups that first colonizing the rumen.

## Conclusions

Our results confirmed a diverse bacterial colonisation from the first day of life in the undeveloped rumen, and show that the colonization pattern substantially differs between pre-ruminants reared under natural or artificial feeding systems. The rumen epithelial immune responded quickly to the early colonization but did not develop differential response to distinct colonizing patterns, suggesting that the niches are open to first colonizers. Longer-term trials are suggested to assess the persistence of differences in adult animals and the metabolic implications.

## Supporting information

S1 Table Primer sequences for toll-like receptors (TLR), β-defensin, peptidoglycan recognition protein 1 (PGLYRP1) and β-actin for real-time PCR. (PDF)

S1 Fig Genus-level taxonomic composition of the ruminal bacterial community at different ages and feeding systems in kids (% of sequences). (TIF)
