# Supplementary material for: Natural and artificial feeding management before weaning promote different rumen microbial colonization but not differences in gene expression levels at the rumen epithelium of newborn goats
Source: PLoS One. 2017 Aug 16;12(8):e0182235. doi: 10.1371/journal.pone.0182235 (PMC5558975; doi:10.1371/journal.pone.0182235)
Supplement: S1 Table — (PDF) [file pone.0182235.s001.pdf]

Supporting Information

S1 Table. Primer sequences for 10 bovine toll-like receptors (TLR), β-defensin, peptidoglycan recognition protein 1 (PGLYRP1) and β-actin used for quantitative real-time PCR

Gene	Forward primer	Reverse primer	Access. No	Product size	Source
**TLR1**	5′CTGCCCATATGCCAAGAGTT3′	5′GGCATCTTCTCTTTCCCCAT3′	AY634628	159	A
**TLR2**	5′CTGTGTGCGTCTTCCTCAGA3′	5′TCAGGGAGCAGAGTAACCAGA3′	AY634629	228	A
**TLR3**	5′TCTTTTCGGGACTGTTGACC3′	5′AAATCCCCCATCCAAGGTAG3′	AJ812026	224	A
**TLR4**	5′GGTTTCCACAAAAGCCGTAA3′	5′AGGACGATGAAGATGATGCC3′	AY634630	137	A
**TLR5**	5′TCAATGGGAGCCAGATTTTC3′	5′CCTTCAGCTCCTGGAGTGTC3′	AY634630	198	A
**TLR6**	5′CGACATTGAAGGCACTGAAA3′	5′TCCTGAGGACAAAGCATGTG3′	AY487803	148	A
**TLR7**	5′TCTCCAAGGTGCTTTCCAGT3′	5′CCACCAGACAAACCACACAG3′	AY487802	166	A
**TLR8**	5′TCACACGGGTAACGAATGAA3′	5′TTTGAGGTTGAGAAATGCCC3′	AY642125	143	A
**TLR9**	5′CTCTCCTTGGACTGCTTTGG3′	5′CACTGCACTCTGCACCTTGT3′	AY859726	204	A
**TLR10**	5′TCACCTGACATCTTTGCGAG3′	5′TCGGAATGGATTTCTTCCTG3′	AY634632	187	A
**β-defensin**	5′GGTCACAAGTGGCAGAGGAT3′	5′TGGTTGAAGAACTTCAGGGC3′	BF775065	152	B
**PGLYRP1**	5′TCCAGCCCCGGCCCTCATAC3′	5′ACTGCGGCAGCATCGTGTCC3′	NM174573	249	C
**β-actin**	5′CTAGGCACCAGGGCGTAATG3′	5′CCACACGGAGCTCGTTGTAG3′	AF191490	177	A

A: Charavaryamath C, Fries P, Gomis S, Bell C, Doig K, Guan LL, Potter A, Napper S, Griebel PJ. Mucosal changes in a long-term bovine intestinal segment model following removal of ingesta and microflora. Gut Microbes, 2011; 2: 134–144.

B: Fjell CJ, Jenssen H, Fries P, Aich P, Griebel P, Hilpert K, Hancock REW, Cherkasov A. Identification of novel host defense peptides and the absence of α-defensins in the bovine genome. Proteins 2008; 73: 420–430.

C: Malmuthuge N, Li M, Fries P, Griebel PJ, Guan LL. Regional and age dependent changes in gene expression of toll-like receptors and key antimicrobial defense molecules throughout the gastrointestinal tract of dairy calves. Vet. Immunol. Immunopathol. 2012; 146: 18–26
