# Supplementary figures and images for: Natural and artificial feeding management before weaning promote different rumen microbial colonization but not differences in gene expression levels at the rumen epithelium of newborn goats
Source: PLoS One. 2017 Aug 16;12(8):e0182235. doi: 10.1371/journal.pone.0182235 (PMC5558975; doi:10.1371/journal.pone.0182235)

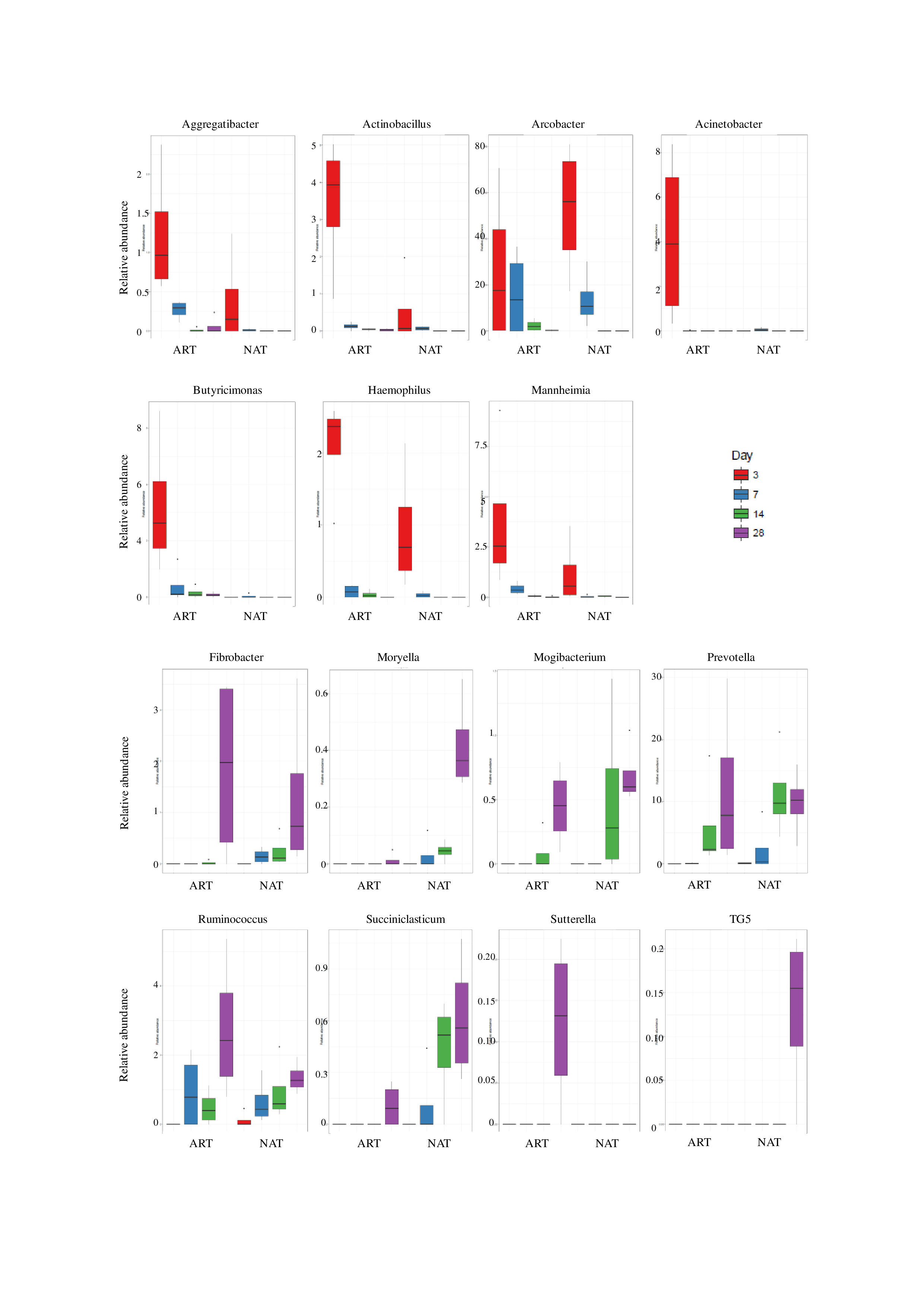

Supplement: S1 Fig — (TIF) [file pone.0182235.s002.tif]
